# Machine Learning-Based Aggression Detection in Children with ADHD Using Sensor-Based Physical Activity Monitoring

**DOI:** 10.3390/s23104949

**Published:** 2023-05-21

**Authors:** Catherine Park, Mohammad Dehghan Rouzi, Md Moin Uddin Atique, M. G. Finco, Ram Kinker Mishra, Griselda Barba-Villalobos, Emily Crossman, Chima Amushie, Jacqueline Nguyen, Chadi Calarge, Bijan Najafi

**Affiliations:** 1 Interdisciplinary Consortium on Advanced Motion Performance (iCAMP), Michael E. DeBakey Department of Surgery, Baylor College of Medicine, Houston, TX 77030, USA; catherine.park@bcm.edu (C.P.); mohammad.dehghanrouzi@bcm.edu (M.D.R.); moindu44@gmail.com (M.M.U.A.); graci.finco@bcm.edu (M.G.F.); ram.mishra@biosensics.com (R.K.M.); 2 Menninger Department of Psychiatry and Behavioral Sciences and Department of Pediatrics, Baylor College of Medicine, Texas Children’s Hospital, Houston, TX 77030, USA; griselda.barbavillalobos@bcm.edu (G.B.-V.); emily.crossman@bcm.edu (E.C.); chima.amushie@bcm.edu (C.A.); jacqueline.nguyen@bcm.edu (J.N.)

**Keywords:** pediatrics, aggression, wearables, remote patient monitoring, machine learning

## Abstract

Aggression in children is highly prevalent and can have devastating consequences, yet there is currently no objective method to track its frequency in daily life. This study aims to investigate the use of wearable-sensor-derived physical activity data and machine learning to objectively identify physical-aggressive incidents in children. Participants (n = 39) aged 7 to 16 years, with and without ADHD, wore a waist-worn activity monitor (ActiGraph, GT3X+) for up to one week, three times over 12 months, while demographic, anthropometric, and clinical data were collected. Machine learning techniques, specifically random forest, were used to analyze patterns that identify physical-aggressive incident with 1-min time resolution. A total of 119 aggression episodes, lasting 7.3 ± 13.1 min for a total of 872 1-min epochs including 132 physical aggression epochs, were collected. The model achieved high precision (80.2%), accuracy (82.0%), recall (85.0%), F1 score (82.4%), and area under the curve (89.3%) to distinguish physical aggression epochs. The sensor-derived feature of vector magnitude (faster triaxial acceleration) was the second contributing feature in the model, and significantly distinguished aggression and non-aggression epochs. If validated in larger samples, this model could provide a practical and efficient solution for remotely detecting and managing aggressive incidents in children.

## 1. Introduction

Aggressive incidents, which range from verbal expressions of anger to physical violence [1], are the most common reason for referral to child and adolescent psychiatric services and the primary indication for inpatient psychiatric admission [2,3,4,5,6,7]. Childhood aggression is often comorbid with other psychiatric disorders, including attention deficit hyperactivity disorder (ADHD), disruptive behavior disorders, mood disorders, anxiety disorders, psychotic disorders, trauma-based disorders, tic disorders, intellectual disability, and autism spectrum disorder [2,3,4,5,6,8]. Aggressive incidents in children have been associated with loss of life, as well as billions of dollars in decreased productivity, quality of life, and healthcare costs [9,10,11].

Despite the high prevalence and devastating consequences of aggressive incidents in children with ADHD, to our knowledge, no objective method exists to track their frequency and severity in daily life. Available tools examine dimensions of aggression (e.g., impulsive vs. premeditated motivations, primary targets, severity, and duration), but they are subjective, typically relying on the parent or caregiver [12,13,14,15]. Consequently, intervention studies targeting aggressive incidents have been largely restricted to rating scales to capture treatment response [16,17,18,19]. While the face validity of the available rating scales is high, accuracy may be suboptimal because informants may have limited reliability. For instance, a parent/caregiver may not be fully informed about the child’s behavior, given the substantial time the child spends at school. A valid and objective method to capture aggressive incidents in daily life could help inform care for children with aggression to ultimately optimize treatment, improve quality of life, and reduce associated costs. Notably, wearable sensors or smart technologies (e.g., smart watches) could offer practical, cost-effective solutions to objectively measure aggressive incidents in children. 

Several studies have demonstrated the efficacy of using wearable sensors to measure physical activity in children with psychopathology. For instance, accelerometers and inertial measurement units have been used to characterize physical activity, sleep patterns, and motor coordination in children with ADHD and other neurological disorders [20,21,22,23,24]. However, these studies focused on task-oriented activities, rather than activities of daily living. Two recent studies used a smartwatch to analyze the movements of children with ADHD at school [25] and while performing daily activities (e.g., sitting, exercises, and household activities) [26]. Notably, Lindhiem et al. used a machine learning approach to establish the context in which hyperactivity is present [26]. As for using wearables specifically to quantify aggressive incidents, Goodwin et al. found that physiological and motion data collected with a biosensor worn by 6 to 17 year old children with autism spectrum disorder admitted to an inpatient psychiatric unit allowed the identification of aggression during observation sessions [27]. However, to our knowledge, this work has not been extended to a naturalistic outpatient setting. 

This study aims to address the existing gap by devising an innovative method for identifying aggressive incidents in children through the use of wearable technology and machine learning. Our objectives are to: (1) assess the efficacy of machine learning in detecting aggressive incidents, (2) uncover distinct physical activity patterns during aggressive incidents, and (3) pinpoint the optimal sensor-driven features, also known as “digital biomarkers,” for identifying aggressive incidents. By utilizing physical activity data gathered from wearable sensors, we hypothesize that our approach will provide a valid and dependable means of detecting aggressive incidents in children.

## 2. Materials and Methods

### 2.1. Participants 

Data for this analysis were collected in the context of a year-long, observational study examining the skeletal effects of psychostimulants. Participants were recruited from pediatric outpatient clinics. Eligible participants were medically healthy males and females who were 7 to 16 years old, of all racial and ethnic backgrounds, within one month of starting a psychostimulant or unmedicated, with no plans to move out of state for one year. Potential participants were excluded if they: (1) had treatment with psychotropics or other medications affecting bone metabolism within the prior year, (2) had serious medical conditions involving a vital organ or affecting bone metabolism, or pregnancy/lactation, (3) were underweight or overweight (i.e., body mass index less than the 5th percentile or greater than the 95th percentile), (4) had a substance use disorder or an eating disorder, or (5) had an intellectual disability or an inability to understand the English language, which would impair their ability to complete study procedures. The local Institutional Review Board approved the study, and written informed consent and verbal assent were obtained.

After completing the baseline visit, participants returned for an in-person visit at 6 and 12 months and completed a monthly phone visit in between. At every contact, the medical and treatment history was updated, including psychostimulant use. In addition, medication adherence was assessed. At in-person visits, demographic and anthropometric variables were collected, including age, sex, height, and weight. The parent also completed the child behavior checklist (CBCL), a widely used rating scale with excellent validity and reliability [28,29]. The CBCL generates a dimensional profile composed of several problem scales, including rule-breaking and aggressive behavior scales. Extensive norms are available, allowing for generation of T-scores. The study psychiatrist also completed the clinical global impression scale (CGI-Severity), a single-item scale with scores ranging between 0 (not assessed) and 7 (among the most extremely ill patients), with a score ≥3 typically considered clinically significant [30]. Clinical diagnoses were based on the Diagnostic and Statistical Manual of Mental Disorders (DSM-5) [31] and incorporated information from the medical record, the rating scales, and an unstructured interview of the parent/guardian and the child completed by a child and adolescent psychiatrist. 

Height and weight were used to calculate body mass index (BMI, kg/m^2^), and sex-age-specific BMI Z-scores were derived [32]. Where applicable, the daily psychostimulant dose was converted to a methylphenidate (MPH) equivalent [33] and the daily average weight- and adherence-adjusted dose in MPH equivalent was computed. The T-score of the CBCL factors was dichotomized based on a threshold of ≥60 to identify participants at risk for clinically significant behavioral problems [29].

Because parents may not be aware of their child’s aggressive incidents on days when school is in session, days when physical activity was collected were classified as “school” vs. “non-school” days. The latter included weekends and holidays.

### 2.2. Assessment of Physical Activity and Aggression Episodes

**Figure 1:**
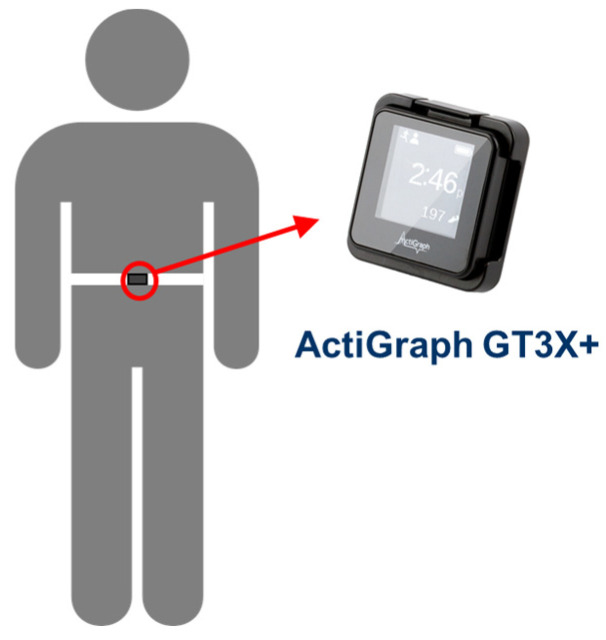
A wearable sensor (GT3X+, ActiGraph Corp., Pensacola, FL, USA) and its placement for physical activity assessments.

At the in-person visits, participants were provided with a waist-worn accelerometer sensor (GT3X+, ActiGraph Corp., Pensacola, FL, USA), which they were instructed to wear for seven consecutive days starting from the day following their visit (Figure 1). The ActiGraph GT3X+ is compact (4.6 cm (W) × 3.3 cm (H) × 1.5 cm (D)), lightweight (19 g), user-friendly, and has a battery life of up to 25 days. In addition, the parents were given a log, based on the Retrospective Modified Overt Aggression Scale (R-MOAS) [34], and asked to record (during the same seven-day period) the date, time, and duration of both verbal-aggressive incidents and physical-aggressive incidents. Given that physical aggression incidents are more problematic and can be reported more validly, this study concentrated on the physical-aggressive incidents, which included incidents towards other people, incidents involving property, and incidents directed at self. 

Information from the R-MOAS allowed definition of the aggression periods, each comprising one or more continuous aggressive incidents. 

### 2.3. Sensor-Drived Features 

To quantify physical activity, seven variables (vector magnitude, cadence, % standing, % sitting, % lying, kilocalories, and MET rate; Table 1) were calculated, using ActiLife software based on 1-min-long epochs (ActiGraph, Pensacola, FL, USA) [35,36]. Vector magnitude indicates the time rate of change of movement speed [37]. Kilocalories indicates the amount of energy relative to body mass and vector magnitude [38]. Metabolic rate (i.e., MET rate) indicates the rate of energy expended per unit of time.

### 2.4. Random Forest Classifier Approach for Feature Selection

**Figure 2:**
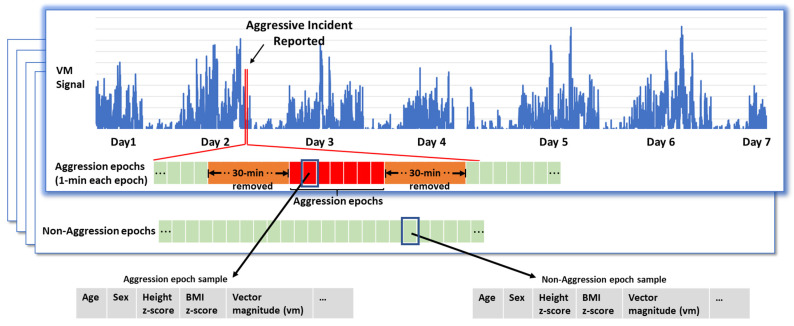
(**Top**): An example of the vector magnitude (vm) signal, which is one of the sensor-derived physical activity variables, for both aggression and non-aggression epochs. Each rectangle represents an epoch, consisting of 1-min worth of physical activity data. The red-filled boxes indicate that the epoch occurred during a reported aggression episode, while the green ones indicate the absence of aggression. The large orange rectangles represent 30-min worth of data occurring before and after an aggressive incident. These were excluded from the analyses to minimize contamination risk. (**Bottom**): This diagram illustrates the different types of variables that are associated with each epoch for inclusion in the model. These variables include demographic, clinical, sensor-derived physical activity, and observed day type variables.

Random forest uses ensemble learning, combining multiple classifiers to provide solutions to complex problems [39,40], and is also commonly used for feature importance determination [39]. As such, our approach used a random forest algorithm both for feature selection and the construction of a classification model, seeking to rank demographic, clinical, sensor-derived physical activity, and observed day type variables with regard to their potential to capture aggressive incidents. To do so, each 1-min epoch was categorized as aggression or non-aggression, based on whether it occurred during a reported time period of aggression (based on the R-MOAS) or not, respectively. All aggression epochs were included in the model. As expected, the number of non-aggression epochs was much larger, causing unbalance in the dataset. To address this issue [41], an equal number of non-aggression epochs was included, selected randomly from all available non-aggression epochs with the following restrictions: (1) non-aggression epochs derived from sleep time (i.e., 10 p.m.–6 a.m.) and (2) those occurring within 30 min before and after an aggression epoch, to minimize any potential contamination effect (Figure 2).

The random forest classifier algorithm included several features, including age, sex, sex-age-specific BMI and height Z-scores, the weight-adherence-adjusted dosage of psychostimulant (in MPH equivalent, mg/kg), whether the epoch occurred on a school day, illness severity based on the CGI, and T-scores for six CBCL factors: total, internalizing, externalizing, attention problems, rule breaking, and aggressive behavior. Additionally, the algorithm considered seven sensor-derived variables (see Table 1). By including both demographic and clinical factors, as well as sensor-derived variables, the algorithm can provide a more nuanced understanding of the relationship between these factors and aggressive incidents. The sensor-derived variables, in particular, are important as they offer an objective measure of physical activity and movement patterns, potentially related to the occurrence of aggressive incidents.

**Figure 3:**
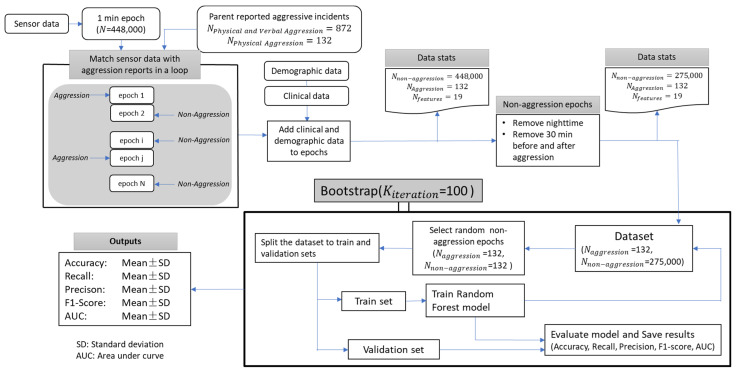
This flowchart illustrates the data preprocess and feature selection using a machine learning model, as well as the evaluation of the resulting model. The random forest classifier model was employed, with 100 bootstraps utilized to assess model performance. The evaluation metrics included mean ± SD values for model accuracy, recall, precision, F1 score, and area under the curve (AUC).

Figure 3 depicts a flowchart divided into two steps. The initial step involved data cleaning, analysis, and preparation for the machine learning model, while the second step detailed the model’s design. In the primary step, sensor data were labeled based on parent-reported physically aggressive incidents, and clinical and demographic information were integrated into each epoch. Subsequently, epochs collected during sleep and within 30 min before and after aggressive incidents were excluded. The second step comprised a two-step feature selection process. The first step was to evaluate the importance of all sensor-derived features using a random forest classifier. We used training and testing sample sizes of 70% and 30%, respectively. The random forest model was trained with 500 trees with a balanced subsample for class weights to improve performance [42,43]. We also used a grid search cross-validation technique to optimize three attributes of the random forest model, an effective approach for optimizing the performance of classifiers [44,45].

In the second step, we ran a random forest method with the same training and testing sizes of 70% and 30%, respectively. This procedure was repeated for 100 bootstraps to ensure that all observations were selected in the validation sub-sample, allowing computation of the means and their standard deviations to quantify their uncertainties [46]. Each step involved adding one feature to the dataset and training the model to gain insight into how adding each feature affects the model’s performance. The final reports comprised the mean and standard deviation (SD) of all 100 estimated model performance metrics, including the accuracy, recall, precision, F1 score, and area under the curve (AUC). Feature selection processes were conducted using Python version 3.10 (Python Software Company, Fredericksburg, VA, USA).

### 2.5. Statistical Analysis

Continuous data are expressed as mean ± standard deviation (SD), while categorical data are reported as count (%). The Shapiro–Wilk test was used to determine whether continuous variables followed a normal distribution. The one-way ANOVA for normally distributed variables and the Mann–Whitney U test for non-normally distributed variables were used to compare mean differences between epochs with vs. those without aggression. The chi-square test was used to examine between-group differences in categorical variables. All statistical analyses were conducted using IBM SPSS Statistics version 27 (IBM Corp., Armonk, NY, USA).

## 3. Results

### 3.1. Demographic and Clinical Characteristics

**Figure 4:**
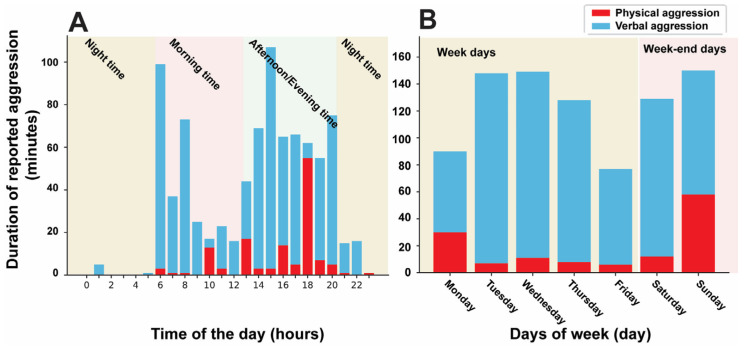
(**A**) The duration of reported aggression (verbal and physical) as a function of time of day. (**B**) The duration of reported aggression (verbal and physical) as a function of weekdays and weekend days. The majority of physical aggression epochs occurred during the afternoon and evening, and on weekend days.

Table 2 presents the demographic and clinical characteristics of participants who had at least one time period of aggression. There were a total of 31 time periods of physical aggression, each lasting an average of 4.3 ± 7.8 min, resulting in 132 physical aggression epochs. Figure 4A depicts the analysis of the duration of reported aggression (verbal and physical) as a function of time of day, including night time (10 p.m.–6 a.m.), morning time (6 a.m.–12 p.m.), and afternoon/evening (12 p.m.–10 p.m.) (time frames selected for purposes of visualization). Time periods of physical aggression mostly occurred after school and were most pronounced around 6 p.m. Time periods of verbal aggression had two pronounced peaks at 6 a.m. and 3 p.m. Furthermore, the majority of physical aggression occurred during weekend days, particularly on Sundays (Figure 4B). 

### 3.2. Difference in Features between Aggression and Non-Aggression Epochs

Table 3 presents descriptive statistics for the physical activity and observed day type features of both aggression and non-aggression epochs collected from 39 participants with ADHD (n = 35) and without (n = 4). Movement acceleration (accelerometer vector magnitude) was significantly higher during the former. 

### 3.3. Optimal Feature Selection and Evaluation 

**Figure 5:**
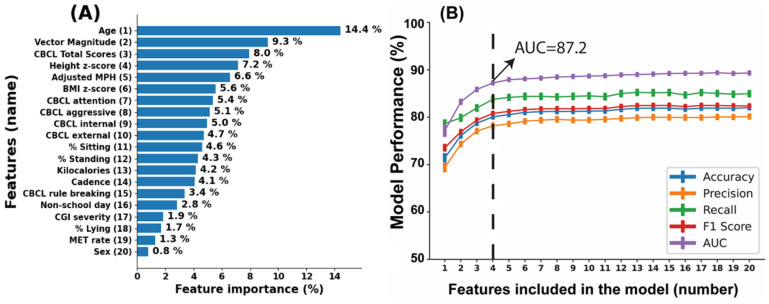
Figure (**A**) shows the ranking of 20 features based on their significance in distinguishing physical aggression from non-aggression epochs, as determined by the random forest classifier algorithm. Meanwhile, Figure (**B**) demonstrates the model’s effectiveness in distinguishing between the two groups, as measured by AUC, F1 score, accuracy, recall, and precision. The figure utilizes several abbreviations, such as CBCL (child behavior checklist), MPH (methylphenidate), CGI-S (clinical global impression-severity), AUC (area under curve).

Figure 5A displays the ranking of the 20 features examined, using the random forest classifier algorithm. A higher percentage value indicates that the feature had a greater contribution to the model. The top 10 features that contributed most to the model, in order of importance, were age (14.41%), vector magnitude (9.27%), CBCL Total T-score (7.96%), height Z-score (7.16%), adjusted MPH dose (6.57%), BMI Z-score (5.56%), CBCL attention T-score (5.37%), CBCL aggressive T-score (5.12%), CBCL internalizing T-score (4.97%), and CBCL externalizing T-score (4.70%).

Figure 5B shows the model validation results as AUC, F1, accuracy, recall, and precision as a function of the number of ranked features. The model with all 20 features achieved an AUC of 89.3 ± 4.2%, F1 of 82.4 ± 4.7%, accuracy of 82.0 ± 4.8%, recall of 85.0 ± 6.6%, and precision of 80.2 ± 5.3%. For the excellent range of AUC and F1 (0.8 to 0.9) and acceptable range of accuracy, recall, and precision (0.7 to 0.8), age, vector magnitude, CBCL Total T-score, and height Z-score were identified as the most important features. A model using these four features achieved an AUC of 87.2 ± 4.1%, F1 score of 80.8 ± 4.3%, accuracy of 80.1 ± 4.6%, recall of 83.8 ± 6.7%, and precision of 78.2 ± 5.9%. When the CBCL Total T-score feature was excluded from the model, the performance decreased by about 10%, resulting in an AUC of 77.9 ± 5.2, an F1 score of 71.8 ± 5.0, an accuracy of 71.6 ± 4.8, a recall of 71.7 ± 8.3, and a precision of 71.9 ± 5.4. However, these values still fall within an acceptable range. It should be noted that the collection of CBCL values is often resource-intensive, and their exclusion may make the model more practical for implementation in certain settings.

## 4. Discussion

This study aimed to investigate whether sensor-derived physical activity data would optimize the identification of aggressive incidents in children, using machine learning. First, this approach achieved a high performance. Second, four features disproportionately accounted for the excellent model performance. Third, three of these top features were objective measures, including age, height, and sensor-based vector magnitude (triaxial acceleration). Finally, excluding the one measure based on parent report (i.e., the CBCL Total Score) reduced model performance, though not drastically. 

Identifying aggression epochs is a crucial step in preventing and managing aggressive incidents. However, unlike the vast majority of the work undertaken so far, which has relied on subjective report, our study suggests that objective assessment is feasible, with potentially high reliability. Importantly, a wearable technology-based measure, specifically vector magnitude, appears to comprise one of the top four features contributing to the model’s excellent performance. This feature has face validity, given that physical aggression often involves rapid triaxial acceleration in order to commit the physically violent act. It, then, follows that tracking vector magnitude could be useful in identifying the onset of aggressive incidents. Such information can aid in developing remote patient monitoring systems that not only may inform parents (or other interested parties, including clinicians) about incidents of aggression in real-time but also potentially predict these incidents. This would allow a more timely intervention to prevent escalation and adjust treatment. 

Although physical activity was the primary focus of our study, demographic and clinical features also played a prominent role in identifying aggression epochs. Our findings showed that age and CBCL Total Score were the first and third most significant feature in our model, respectively. Although the training set of physical aggression epochs and non-aggression epochs were randomly selected from the data pool of all aggression and non-aggression epochs, the average age and CBCL Total Score for selected physical aggression epochs were 8.5 ± 1.3 years and 43.6 ± 29.5, respectively. In contrast, these numbers were 9.5 ± 1.7 years and 28.2 ± 49.0 for the randomly selected non-aggression epochs, indicating a significant difference between physical aggression and non-aggression epochs (*p* < 0.0001). Younger age was the strongest feature in the model associated with aggression epochs. This was consistent with well-established findings of reduced aggression with development and maturation of inhibitory and self-regulation processes [47,48,49,50]. These two objective features are relatively easy to document reliably and appear to substantially contribute to a model. 

Of the top four features contributing to the model’s performance, only the CBCL score is based on subjective report. As would be expected, a higher score (denoting more severe psychiatric difficulties) is associated with a greater likelihood of physical aggressive incidents [51]. The CBCL is based on parental report and has excellent psychometric properties. It is a “broad-band” measure that captures various aspects of psychopathology, over the prior 6-month period. As such, it is quite distinct from many subjective measures of aggression, that are more directly temporally linked to such incidents. In other words, while the CBCL has the shortcoming of being subjective, it provides the necessary context within which to “interpret” an epoch as aggression or non-aggression.

In our study, of the 20 features considered in our model, sex had the least impact on its performance. This might appear surprising at first glance, given the well-established preponderance of aggression among boys. However, it is likely that the effect of sex was mediated by other variable including height, acceleration, overall activity level, etc. 

We observed that the majority of physical aggression episodes occurred during the afternoon and evening, and on weekend days. This finding was consistent with previous research indicating that physical aggression among preschool-aged children is more likely to occur in the late afternoon and early evening hours and on weekends compared with weekdays [49]. One potential explanation is that these are the times when the children are with their parents or caregivers who are serving as the informants. This underscores the importance of optimizing the process to capture problematic behavior by making it less informant-dependent. Other potential factors explaining the higher likelihood of physical aggression during the afternoon, evening, and weekends include poorly controlled ADHD symptoms, increased free time, reduced supervision, social and peer pressure, and fatigue or stress [52,53].

It is important to note that the findings of this study do not suggest that children who exhibit the identified features are more likely to have aggressive incidents. Rather, these features may serve as potential indicators for identifying such episodes. To establish a causal association between these features and the prevalence of aggressive incidents, interventional studies are needed. Potential applications of these findings in clinical practice may include exporting physical activity data from smartphones or wearable sensors during clinical visits, with a particular focus on technology that exports vector magnitude. Additionally, a clinical tool could be developed to remotely track aggressive incidents, especially during the school day when parents/caregivers may not be aware of their occurrence. 

While to our knowledge this is the first study to examine aggressive incidents in children in a real-life setting, several limitations should be acknowledged. Results may be skewed by a few participants with significantly longer time periods of physical aggression compared with other participants. To address this limitation, we performed a secondary analysis and down-sampled the number of epochs from participants who had considerably longer durations of physical aggression epochs compared with other participants. We identified one participant with a very long duration of a physically aggressive event (45 min). When the number of epochs for this subject was down-sampled and the model was fitted again, the model’s performance was minimally impacted (precision = 76.4 ± 5.5% [decreased by 3.8%], accuracy = 78.6 ± 4.9% [decreased by 3.4%], recall = 83.9 ± 8.4% [decreased by 1.1%], F1 score = 79.7 ± 4.9% [decreased by 2.7%], and area under the curve = 85.7 ± 5.4% [decreased by 3.6%]). Nonetheless, our findings should be confirmed in a larger sample.

ADHD is a well-known risk factor for aggression but neither the presence of ADHD nor of aggression was required for study participation. As such, future studies should include children exhibiting higher aggression severity to confirm the generalizability of our findings. In the current study, similar to others [39,40], our approach used a random forest classifier to construct a classification model. Additionally, we conducted a secondary analysis to demonstrate the relative importance of each feature. Although we assessed the optimized feature selection through cross-validation and bootstrapping, further validation using an independent dataset is necessary to confirm our findings and ensure their robustness and generalizability. Moreover, future work with larger sample sizes must explore other machine learning techniques and approaches, including innovative models seeking to better capture complex human behavior [54]. Another limitation is that aggressive incidents were recorded by parents/caregivers using a paper log, with instructions to record the events promptly. Nonetheless, their report may have been subject to recall bias or inaccuracy. To address this limitation, future studies could include teacher informants and/or an online platform to immediately report aggressive incidents in a more timely and accurate manner. Moreover, ensuring consistent sensor use among children displaying aggressive behavior may be challenging. Practical limitations, including restricted battery life and discomfort from extended wear, also pose difficulties. To bolster the effectiveness and broad adoption of our approach, future research should investigate complementary solutions such as video tracking, e-tattoo sensors, textile sensors integrated into clothing (e.g., smart t-shirts), and alternative sensor designs such as pendant sensors, which might be more acceptable to the target population. Future improvement should also include providing real-life alerts to caregivers when an aggressive episode occurs. Our study could not differentiate verbal aggressive behavior. Although most verbal aggression may be accompanied by physical agitation, relying solely on accelerometer sensors may not be adequate for identifying such behavior. Future research should explore the use of complementary sensors capable of capturing voice and volume to better detect verbal aggressive behavior. Finally, future studies could develop a mathematical model to detect aggressive behavior with a higher accuracy and determine the relationship between time of medication use and aggressive incidents reported in the evening. 

## 5. Conclusions

This study presents a promising approach to identify incidents of physical aggression in children, using physical activity monitoring and machine learning. Faster movement speed, measured by vector magnitude, in combination with age, height, and CBCL Total Score could help identify physically aggressive incidents. Further research is needed to confirm the validity of these findings and explore clinical applications, including for prevention or treatment adjustment. For example, identifying predictors of aggression may help in the timely deployment of de-escalation procedures. Finally, given known racial/ethnic inequities in responding to problematic behavior [55], a tool to capture aggression objectively may reduce structural racism in healthcare and educational settings. 

## Figures and Tables

**Table 1:** Sensor-derived physical activity variables, measured in 1-min epochs. The physical activity variables are obtained from a waist-worn accelerometer and reflect the intensity and level of physical activity in 1-min intervals. These variables are utilized to identify and describe aggression epochs, and they are also one of the inputs to the machine learning model.

Name	Unit	Description
Vector magnitude (vm)	cpm	Total vector magnitude of three accelerometer axes, calculated by the ActiGraph using the following equation [37]: $\mathrm{vm}=\sqrt{axis1^{2}+axis2^{2}+axis3^{2}\;}$
Cadence	steps/min	Step counts per minute
% Standing	% of epoch	Percentage of an epoch when participant was standing
% Sitting	% of epoch	Percentage of an epoch when participant was sitting
% Lying	% of epoch	Percentage of an epoch when participant was lying
Kilocalories	cpm	Kilocalories expended per minute, calculated by the Actigraph using the following equation [38]: $\frac{\mathrm{kcal}}{\min}=\left(0.001064\times \mathrm{vm}\right)+\left(0.087512\times \mathrm{body}\;\mathrm{mass}\;\mathrm{in}\;\mathrm{kg}\right)-5.500229$
MET Rate	cpm	Metabolic equivalents to measure energy expenditure

cpm: counts per minute. MET: metabolic rate.

**Table 2:** Demographic and clinical characteristics.

Variables	N = 39
**Demographics**	
Age, years	9.4 ± 1.7
Sex (Male), n (%)	31 (79.5%)
Hispanic, n (%)	13 (33.3%)
Race, (%)	
White	29 (74.4%)
Black	5 (12.8%)
Native American	1 (2.6%)
Others	4 (10.3%)
Height (cm)	136.2 ± 12.6
Height Z-score	0.008 ± 0.893
Weight (kg)	32.1 ± 9.2
Body Mass Index, kg/m^2^	17.0 ± 1.9
Body Mass Index Z-score	0.1 ± 0.8
**Clinical Characteristics**	
Medication, N (%)	34 (87.2%)
Daily Average Weight and Adherence Adjusted Dose in MPH Equivalency (MPH Eq mg/kg)	0.51 ± 0.37
CBCL, Total Score	44.2 ± 10.4
Aggressive Behavior	60.7 ± 9.1
Rule-Breaking Behavior	57.4 ± 7.6
Attention Problems	64.1 ± 9.1
Internalizing Problems	54.1 ± 12.1
Externalizing Problems	58.1 ± 10.9
CGI-Severity Score	4.7 ± 1.0

Values are presented as mean ± standard deviation or n (%). All CBCL components are reported in T-Scores. MPH: methylphenidate; CBCL: child behavior checklist; CGI-Severity: clinical global impression-severity.

**Table 3:** Comparison of sensor-derived body motion and posture characteristics and observed day type between physical aggression and non-aggression epochs. The term “non-aggression epochs” represents periods where no physical aggressive incident was observed, while “aggression epochs” refers to the time periods during which a physical-aggressive incident was recorded. The table displays the mean values and standard deviations of each variable for both aggression and non-aggression epochs, as well as the *p*-value from the statistical test of the difference between the two groups.

Variables	Aggression Epochs N = 132	Non-Aggression Epochs N = 132	*p*-Value
**Physical activity**			
Vector magnitude (vm)	1580.7 ± 1831.1	873.3 ± 1137.2	0.027 *
Cadence (cpm)	15.3 ± 22.5	8.1 ± 13.6	0.102
% Standing	51.4 ± 47.7	44.6 ± 46.2	0.320
% Sitting	16.9 ± 34.2	26.4 ± 41.9	0.176
% Lying	15.7 ± 36.0	14.9 ± 35.7	0.478
Kilocalories (cpm)	0.27 ± 0.46	0.17 ± 0.23	0.427
MET rate (cpm)	1.15 ± 0.43	1.04 ± 0.12	0.132
**Observed day type**			
Non-school day	47.0%	47.7%	0.902

Values are presented as mean ± standard deviation or n (%). Asterisk denotes a significant difference between the groups. cpm: counts per minute. MET: metabolic rate.

## Data Availability

The de-identified datasets (except sensor data) are available upon request to the corresponding author(s).
